# Short-term clinical outcomes after off-pump coronary artery bypass grafting at a single Veterans Affairs Medical Center

**DOI:** 10.1186/s13019-016-0480-5

**Published:** 2016-05-17

**Authors:** Entela B. Lushaj, Athanasia Schreiner, Besa Jonuzi, Abbasali Badami, Nilto DeOliveira, Lucian Lozonschi

**Affiliations:** Department of Surgery, Division of Cardiothoracic Surgery, University of Wisconsin School of Medicine and Public Health, 600 Highland Avenue, Madison, WI 53792 USA; William S. Middleton Veterans Hospital, Madison, Wisconsin USA

**Keywords:** CABG, Off-Pump, Outcomes, Veterans Affairs

## Abstract

**Background:**

We retrospectively assessed the outcomes after coronary revascularization at a single Veterans Affairs Medical Center when a strategy of assigning higher risk patients to off-pump coronary artery bypass grafting (CABG) was employed.

**Methods:**

Over a 5 year period all consecutive patients that underwent CABG at our VA Medical Center were assigned to a surgeon who either performs the CABG exclusively off-pump or to one who performed the CABG on-pump. The higher risk patients were assigned preferentially for off-pump revascularization. VASQIP (VA Hospitals Surgical Quality Improvement Program) data between 10/2007 and 12/2012 were retrospectively reviewed at our VA Medical Center and the short term outcomes were assessed.

**Results:**

A total of 252 consecutive patients underwent off-pump CABG (*n* = 170) and on-pump CABG (*n* = 82). There were significantly more patients with low LVEF (<45 %; *p* = 0.008) and cerebrovascular disease in the off-pump group (*p* = 0.024). The number of patients smoking at the time of surgery was significantly higher in the off-pump group (*p* = 0.002) as well. The 30-day composite morbidity and mortality was 6 % for all CABG patients and significantly lower with off-pump *vs*. on-pump CABG (3.5 % *vs*. 11 %; *p* = 0.019). There were no conversions from off-pump to on-pump surgery.

**Conclusions:**

A selective strategy to direct higher risk patients towards an off-pump revascularization yielded favorable outcomes in an unselected veteran population treated at a single VA Medical Center over a 5 year period.

## Background

The benefits of off-pump coronary artery bypass grafting (CABG) in low risk patients remains debatable and still unknown since very large randomized trials would be required to show a difference in stroke and mortality when compared to on-pump CABG. Nevertheless, there are numerous studies that show benefits with the off-pump revascularization in low as well as high risk patients [1–5]. The former showed shorter intensive care unit (ICU) and hospital stay, shorter ventilation time, decreased rate of atrial fibrillation, transfusion, inotrope requirements, respiratory infections, stroke, delirium, and postoperative MI [1]. Other studies reported that the off-pump CABG has no advantages or even disadvantages over on-pump, such as equal mortality, myocardial infarction, stroke, equal or lower long-term graft patency [6–9].

The purpose of the present study was to compare short-term outcomes (mortality and complications) for all consecutive patients undergoing CABG surgery at our Veterans Affairs (VA) center from 2007 to 2012. Over this five year period, we have used a selective approach in directing higher risk patients towards off-pump coronary artery bypass surgery with the aim of improving overall outcomes.

## Methods

We retrospectively studied patients that underwent a CABG procedure at our VA Hospital, between October 2007, and December 2012. A strategy to assign higher risk patients for off-pump revascularization was employed, while lower risk patients were randomly assigned to receive CABG either on pump or off-pump. The procedures were performed by two experienced surgeons who performed the procedure either 100 % off-pump or on-pump, respectively. There were no patient exclusions. In this study, labeling a patient at higher postoperative risk was a clinical decision made by the surgical team when one or more co-morbidities perceived to increase the risk of postoperative complications were identified during standard preoperative work-up. Proximal anastomoses were performed using a no-clamp technique for the aorta employing an anastomotic enabling device (HEARTSTRING System: MAQUET, San Jose, CA, USA) in 100 % of cases. Short-term clinical outcomes including 30-days operative mortality and perioperative morbidity were evaluated. The 30-day operative mortality was defined as any death occurring during the index hospitalization or within 30 days after surgery. The perioperative morbidity was defined as the presence or absence of any of the following major complications: cardiac arrest, renal failure requiring dialysis, stroke (acute loss of focal neurological function lasting >72 hours with our without permanent neurological deficit), coma, and repeat cardiac surgery, reoperation for bleeding, new mechanical support, mediastinitis and tracheostomy during the inpatient perioperative period or within 30 days after surgery.

### Statistical analysis

Continuous variables are presented as mean ± standard deviation, and categorical variables are represented as number and percentage. When the distribution appeared to be skewed we produced median and interquartile ranges (IQ) ranges and compared using the Mann-Whitney U-test. Continuous variables were compared using t-test. Categorical variables were compared using the chi-square test. *P*-values less than 0.05 (two-sided) were considered statistically significant. All analyses were performed using the IBM SPSS statistical software program (IBM SPSS Statistics for Windows, Version 22.0. Armonk, NY: IBM Corp).

## Results

### Demographic profiles

Two hundred fifty two patients underwent a CABG procedure from 10/2007 through 12/2012. One hundred seventy (67 %) of those procedures were performed off-pump and 82 (33 %) were performed on-pump. Patient demographics and preoperative characteristics are shown in Table 1. Mean age for both off- and on-pump CABG patients was similar (*p* = 0.84). The majority of the patients in both groups were males with a mean BMI of 31 kg/m^2^. All of the cases were performed as elective procedures and none of the patients had a previous cardiac surgery. There were significantly more patients that were active smokers (*p* = 0.002), with low LVEF (<45 %; *p* = 0.008) and cerebrovascular disease (CVD; *p* = 0.024) in the off-pump CABG *vs*. on-pump CABG groups (Table 1). The number of patients with COPD, diabetes, hypertension or peripheral vascular disease was similar in both groups (Table 1).

**Table 1:** Pre-operative patient background

	Off-pump (*n* = 170)	On-pump (*n* = 82)	*p*-value
Demographics			
Age (years)	64 ± 9	65 ± 8	0.84
Gender (male)	169 (99 %)	80 (98 %)	0.51
BMI (kg/m^2^)	31 ± 6	31 ± 6	0.81
Urgent Status	18 (11 %)	15 (18 %)	0.12
Previous heart surgery	0	0	
Current smoker	48 (28 %)	9 (11 %)	0.002*
Comorbidities			
COPD	43 (25 %)	14 (17 %)	0.15
PVD	26 (15 %)	8 (10 %)	0.27
DM	70 (41 %)	38 (46 %)	0.45
HTN	161 (95 %)	74 (90 %)	0.13
CVD	48 (28 %)	12 (15 %)	0.024*
Cardiac function			
LVEF < 45 %	47 (28 %)	11 (13 %)	0.008*

Values are n (%) or mean ± SD. *Statistically significant associations (*p* < 0.05). *BMI* body mass index; *COPD* chronic pulmonary obstructive disease; *PVD* peripheral vascular disease; *DM* diabetes mellitus; *HTN* hypertension; *LVEF* left ventricular ejection fraction; *CVD* cerebrovascular disease

### Post-operative outcomes and complications

Both off-pump and on-pump patients had similar number of grafts (3 ± 1; *p* = 0.99). None of the patients who underwent off-pump CABG were converted to on-pump during surgery (Table 2). While the off-pump CABG patients had a higher ventilation time (7.4 ± 1.1 *vs*. 6.5 ± 0.1; *p* < 0.001), intensive care unit (ICU) and hospital length of stay was similar in both groups (Table 2). None of the off-pump patients had a stroke or coma (Table 3). Off-pump CABG patients had a significant lower 30-day composite risk of morbidity and mortality (*p* = 0.019). Other short-term end points such as, 30-day mortality, the number of bypass grafts, rate of infections or reoperations for bleeding were not significant between the 2 groups (Table 3). In this study NMS consisted of new IABP only, no patients required ECMO or VAD support.

**Table 2:** Clinical Outcomes

	Off Pump (*n* = 170)	On-Pump (*n* = 82)	*p*-value
Number of grafts	3 ± 1	3 ± 1	0.99
Conversion to On-pump	0	NA	
Ventilation time (h)	7.4 ± 1.1	6.5 ± 0.1	<0.001*
Length of ICU stay (d)	2.9 ± 2.0	2.4 ± 2.5	0.10
Length of hospital stay (d)	9.8 ± 6.5	8.5 ± 5.0	0.08

Values are mean ± SD. *Statistically significant associations (*p* < 0.05). *ICU* intensive care unit

**Table 3:** Post-operative complications

	All patients (*n* = 252)	Off-Pump (*n* = 170)	On-pump (*n* = 82)	*P*-value
30-day composite	15 (6 %)	6 (3.5 %)	9 (11 %)	0.019*
30-day mortality	5 (1.9 %)	3 (1.7 %)	2 (2.4 %)	0.70
Cardiac arrest	1 (0.4 %)	1 (0.6 %)	0	0.49
Renal failure requiring dialysis	1 (0.4 %)	1 (0.6 %)	0	0.49
Stroke	2 (0.8 %)	0	2 (2.4 %)	0.044*
Coma	1 (0.4 %)	0	1 (1.2 %)	0.15
Bleeding requiring reoperation	2 (0.8 %)	1 (0.6 %)	1 (1.2 %)	0.60
Tracheostomy	1 (0.4 %)	1 (0.6 %)	0	0.49
Mediastinitis	6 (2.4 %)	6 (3.5 %)	0	0.088
New mechanical support	3 (1 %)	0	3 (3.6 %)	0.013*

30-day Composite – Death, Reoperation, New mechanical support, Cardiac arrest, Coma, Stroke, or Renal failure

## Discussion

Using a selective strategy to assign higher risk patients for off-pump revascularization, the veterans who received CABG (either on- or off-pump) at our VA Medical Center had a 6 % 30-day composite rate of morbidity and mortality. Cornwell et al [10] reported a 30-day composite rate of 10.5 % in 31942 veterans who underwent CABG between 2004 and 2011 at 42 VA cardiac surgery centers that participate in VASQIP data base.

The ROOBY trial [11] reviewed and compared the short-term and 1-year composite outcomes of patients who received off-pump or on-pump CABG from 18 VA medical centers. There was no significant difference between off-pump CABG and on-pump CABG in the rate of 30-day composite outcome and the rate of 1-year composite outcome was even higher for off-pump CABG than on-pump CABG. The long-term grafts patency was lower and more patients received fewer grafts than originally planned in off-pump CABG in this trial and there were no treatment-based differences in neuropsychological outcomes or short-term use of major resources. ROOBY trial concluded that off-pump CABG offered no benefit over on-pump CABG and resulted in worse composite morbidity and mortality outcomes. We believe the high conversion rates (12 %) in the ROOBY trial [11] and other trials (7.9 % in CORONARY [12] and 5 % in GOPCABE [13] may explain the inferior outcomes with off-pump revascularization in those studies.

When compared with off-pump patients in the ROOBY trial our off-pump CABG patients had lower rates in terms of 30-day composite outcome (7 % vs. 3.5 %), cardiac arrest (1.8 % vs. 0.6 %), stroke (1.3 % vs. 0 %) and reoperation for bleeding (2.7 % vs. 0.6 %). Those patients also had no conversions to on-pump CABG, shorter postoperative stay in the ICU, and shorter total hours on ventilator when compared with patients undergoing off-pump revascularization included in the ROOBY trial [11].

The main limitations of our study include its retrospective nonrandomized design, analysis of limited number of patients from a single institution and the majority of patients being male data because of the nature of the veteran population.

## Conclusions

In conclusion, our strategy to preferentially assign all veterans in need of elective CABG but perceived to have higher risk of perioperative complications for off-pump revascularization resulted in a lower 30-day composite morbidity and mortality in veterans who received CABG at our medical center over a five year period compared to the national average over a similar period of time [10]. Despite having higher prevalence of lower EF, CVD and smoking, veterans who underwent off-pump revascularization had lower short-term composite complication rate than those who underwent on-pump revascularization as well. Higher risk VA patients may benefit from an off-pump approach when CABG is indicated.
